# Nutritional status of children under five years old involved in a seasonal malaria chemoprevention study in the Nanyumbu and Masasi districts in Tanzania

**DOI:** 10.1371/journal.pone.0267670

**Published:** 2022-04-29

**Authors:** Bruno P. Mmbando, Richard O. Mwaiswelo, Frank Chacky, Fabrizio Molteni, Ally Mohamed, Samwel Lazaro, Billy Ngasala

**Affiliations:** 1 National Institute for Medical Research, Tanga Centre, Tanga, Tanzania; 2 Department of Microbiology, Immunology and Parasitology, Hubert Kairuki Memorial University, Dar es Salaam, Tanzania; 3 National Malaria Control Programme, Ministry of Health, Dodoma, Tanzania; 4 Department of Parasitology and Medical Entomology, Muhimbili University of Health and Allied Sciences, Dar es Salaam, Tanzania; UCSI University, MALAYSIA

## Abstract

**Background:**

Malnutrition and malaria are common co-morbidities in low-income countries, especially among under-fives children. But the malnutrition situation in Masasi and Nanyumbu districts, its interaction with malaria infection and the influence of socioeconomic factors are not well understood.

**Methods:**

Children aged between 3–59 months in Masasi and Nanyumbu were screened for nutritional status and malaria infection in the community. Nutritional status was determined using age and anthropometric parameters. Z-scores (weight for age (WAZ), height for age (HAZ) and weight for height (WHZ)) were calculated based on the World Health Organisation (WHO) growth reference curves. Malaria infection was determined using malaria rapid diagnostic test and microscopy. Hemoglobin concentration was assessed using HemoCue spectrophotometer, and anemia was classified as hemoglobin concentration < 11.0g/dL. Structured questionnaire was used to collect socio- demographic information electronically.

**Results:**

A total of 2242 children, 1539 (68.6%) from Masasi and 1169 (52.1%) females were involved in the study. The mean z-scores (WAZ = -0.60 and HAZ = -1.56) were lower than the WHO reference population. The overall prevalence of malnutrition was 49%, and it was significantly higher in Nanyumbu (52.5%) than in Masasi (47.3%), (*x2* = 5.045, *p* = 0.025). Prevalence of malnutrition was higher in boys (53.0%) than in girls (45.0%) (*x*2 = 13.9, *p* < 0.001). Stunting was the most prevalent component of undernutrition; it was slightly prevalent in Nanyumbu (46.5%) compared to Masasi (42.0%), (*x*2 = 3.624, *p* = 0.057) and in boys (48.2%) than in girls (39.1%), *x*2 = 17.44, *p*<0.001. Only 15.8% of the undernourished children had malaria infection. Sex, age group and anaemia were significantly associated with undernourishment (p<0.05), while district and malaria infection were marginally (p≤0.06) associated with undernourishment. None of the undernutrition indices was associated with malaria infection.

**Conclusion:**

Undernutrition was highly prevalent in the study population and was influenced sex, age, anaemia and malaria infection. More emphasis is needed to address the malnutrition problem especially stunting in Masasi and Nanyumbu districts.

## Background

Malnutrition and malaria are conditions of major global public health importance, affecting especially under-five children [1–5]. Malnutrition and malaria independently contribute to a substantial proportion of morbidity and mortality in the age group [6]. Malnutrition, has multiple causes including poverty, food insecurity, lack of diversity in diet, infections, poor access to clean water and inadequate sanitation and hygiene [7]. Malaria, on the other hand, is an infectious disease caused by *Plasmodium* parasites [8,9]. But the fundamental relationship between malnutrition and malaria is complicated [4,6], as there is no consistent association between them [6].

Malaria infections lead to individual energy loss with reduced productivity, and in a long run lead to a spiral of malnutrition and poverty [10,11]. Metabolic activities of the parasite and host also increase the demand for nutrients [10], and the disease accompanying clinical conditions such as intestinal malabsorption and diarrhea perpetuate malnutrition [11]. Malnutrition on the other hand, suppresses the immunity [10,11], and in severe cases both acquired and innate immunity are impaired [10,12]. This immunodeficiency impairs ability to mount effective immune response [11–13] and also increases susceptibility to infections [10]. But whereas some studies indicate malnutrition to increase susceptibility to malaria [14,15], others found malaria to increase the risk of malnutrition [6], and other studies have found no association between the two conditions [6], while others have found malnutrition to be protective against malaria [6,16]. For instance, studies have found a significant increase in risk of malaria infection in stunted [1,3,17,18], but not in wasted [1,3], and underweight children [3]. Parasite density is also found to be higher in wasted than in stunted, underweight and normal children, whereas clinical malaria is higher in stunted [1,19], and underweight children [1,12]. Anemia prevalence is also higher in stunted and underweight children [14,19]. Undernutrition also leads to poor malaria outcomes [20], including an increased death risk [21]. On the contrary, other studies have found stunting [14,16,22], underweight [16,17,22,23], and wasting [16,17,23] to have no association with increased risk of malaria infection. Instead, one study has found a protective effect of stunting against *falciparum* malaria [13], although another study found no protective effect of undernutrition against malaria [16]. Likewise, whereas one study found malaria to have no effect on nutritional status [17], others have found the infection to predispose individuals to malnutrition [5,24]. However, the situation of malnutrition in Masasi and Nanyumbu is not well known, and it is not clear how the two conditions interact to influence one another in the districts.

Importantly, socioeconomic factors that influence malnutrition also influence malaria infection. Indeed, these factors probably modulate the way the two conditions interact to the extent that it is difficult to see a consistent association between them. For instance, studies have found that living in rural areas and low economic status [3,5,15,25–27], lack of formal education of caregivers [3,15,28], gender of child especially male [1,3,5,15,26,27], source of water for domestic use and its distance [27,29], extended larger families [26], occupation of the mother [5,15,26,28], and floor of the house [25] to influence both malnutrition and malaria infection in under-five children. It is however not clear what are the socioeconomic factors influencing malnutrition that may modulate the interaction between malnutrition and malaria infection in Masasi and Nanyumbu districts. This study therefore, intended to assess the situation of malnutrition in under-five children in Masasi and Nanyumbu districts, how it interacts with malaria, and the influence of socioeconomic factors on the condition.

## Material and methods

### Study area

The study was conducted in Nanyumbu and Masasi districts, Mtwara region. Nanyumbu is divided into 14 wards and 89 villages, whereas Masasi is divided into 22 wards and 159 villages.

Nanyumbu has a total population of 166,277, and Masasi has 269,590. The districts had a projected population of 82,740 children aged 3–59 months in 2019, of which 31,564 were in Nanyumbu and 51,176 in Masasi district. Subsistence farming, petty trade, fishing, and small- scale mining are the major economic activities in both districts.

The districts have annual rainfall averaging 939mm and temperature of 25.4°C. The rainy season is between January and April, and accounts for > 60% of the average annual rainfall. Both districts have high seasonal malaria transmissions where > 60% of cases occur between March and July. *P*. *falciparum* is the predominant malaria parasite, and *Anopheles arabiensis* the major vector. Insecticide treated bed-nets and, diagnosis and treatment with antimalarial drugs are the major malaria control measures in the area. The prevalence of stunting in children below 5 years of age in Tanzania is 31.8%, and severe stunting is 10.8%, whereas that of underweight is 14.6% [30]. Mtwara region has a stunting prevalence of 29.6%, and that of underweight is 10.7% [30]. The incidence rate of poverty ranges from 31–37% and 38–67% in Masasi and Nanyumbu district, respectively [31]. In Mtwara region the use of improved toilet facility not shared is at 21.5%, and that of shared is 5.8%, whereas that of unimproved toilet/public toilet is at 72.7% [30].

### Study design

This was a community-based cross-sectional survey with wards used as sampling units. Health facilities nearest to the centroid of the selected wards were identified (index facilities) and the catchment population estimated. A study village(s) within the health facility catchment area was identified (index village(s) and demographic survey was carried out to determine the population structure and the number of possible study children. After that, malariometric, household socioeconomic, and nutritional surveys were conducted in all the selected villages.

### Study population

Children aged 3–59 months were involved in the malariometric and nutritional surveys, where household heads were involved in the household survey. The inclusion criteria for malariometric and nutritional surveys also included living within the catchment area and willingness to participate in the study. The exclusion criteria were presence of severe illness, history of intake of antimalarial drug within the previous 30 days and being under cotrimoxazole prophylaxis [32].

Inclusion criteria for the household survey included living within the catchment area, willingness to participate in the study, and having a child who has participated in the malariometric and nutrition surveys. The head of the household was defined as a person who is perceived by household members to be the primary decision maker in the family, and the household was defined as individuals living together and taking meals from a common cooking facility [33]. In the absence of the head of the household, a responsible person above 18 years was interviewed.

### Procedures

#### Malariometric and nutritional survey

The survey was carried out at the index health facilities whereby all the children of the required age were brought for clinical and laboratory assessments. The survey was conducted for three days at each ward.

Clinical assessment involved taking history of clinical symptoms, history of antimalarial drugs consumption within past 30 days, use of cotrimoxazole as prophylaxis, clinical examination including measurement of axillary temperature and anthropometric measurements. The anthropometric measurements including height/length, weight and mid upper arm circumference (MUAC) were recorded for each child by the same investigator using standard techniques. Height was recorded to the nearest 0.1 cm using anthropometric height rod. For children below 24 months of age, length was measured using infant-meter. Weight measurements were recorded to the nearest 100g using SECA electronic weighing scale. Repeated measurements were made for 20 children to periodically correct for the intra observer error. The z-scores (weight-for-age (WAZ), height-for-age (HAZ) and weight- for-height (WHZ)) were then calculated using the WHO2006 growth reference standards [34]. Nutritional statuses were defined as underweight if WAZ < - 2 standard deviation (SD), stunting if HAZ < -2SD, wasting if WHZ < -2SD or MUAC < 115, thin if BAZ < -2SD and obese if BAZ >2SD. A child was considered undernourished if either was stunted, wasted or underweight [34].

Laboratory assessment involved collection of finger-prick blood samples that were used to determine presence of malaria infection using malaria rapid diagnostic test (mRDT), prepare thick film for microscopy to assess density of asexual parasitemia and gametocytemia, and thin film for asexual parasite species determination. The blood samples were also used to measure haemoglobin concentration.

Thin films were fixed using methanol. Both thin and thick films were air-dried, stained using 3% Giemsa stain for 1 hour, and examined for malaria parasites at 100 high power fields under immersion oil. Parasitemia was determined by counting the number of parasites present per 200 white blood cells (WBC) on a thick smear, and the number obtained was multiplied by 40 assuming a WBC count of 8,000 per milliliter of blood. If no parasite was seen after examining 100 fields, then a blood slide was considered negative. Two independent laboratory technicians who were unaware of the mRDT results read the slides. In case of a discrepancy (positive versus negative or a difference in parasite density greater than 30%), a third reading was requested and the average parasite density of two or three readings was used. Microscopists at the National Institute for Medical Research (NIMR) read 10% of the slides for quality control.

A portable spectrophotometer, HemoCue Hb 301+ (HemoCue AB, Ängelholm Sweden) with a precision of +/- 0.3g/dL was used to measure hemoglobin concentration. The HemoCue was calibrated every morning using a control cuvette at 16.0+/-0.3g/dL according to manufacturer’s instruction. Anaemia was classified as hemoglobin level < 11g/dL [35].

### Socioeconomic survey

It was carried out in catchment villages of the selected wards. Community health workers (CHWs) conducted the household survey using a structured questionnaire with both close and open ended questions. The questionnaire inquired information on demographic characteristics of the household, socioeconomic status including building materials used in the main house (type of roof, type of window, type of floor, type of walls), occupation of head of household and household assets such as ownership of radio, bicycle and mobile phone, ratio of number of sleeping bedrooms to number of household dwellers, type and number of animals, source of power for lighting and cooking, and size of cultivated land owned by the family. These variables were selected as a proxy for socio-economic status because is difficult to obtain household income data [36].

### Study outcomes

The primary outcome was the prevalence of malnutrition in under-five children surveyed. Secondary outcomes include: i) prevalence of any indicator of malnutrition, ii) prevalence of malaria infection, iii) prevalence of anaemia defined as a haemoglobin concentration of less than 11g/dL, (iv) prevalence of socioeconomic factors influencing malaria transmission and malnutrition.

### Ethical consideration

The study was conducted in accordance with the declaration of Helsinki, good clinical practices, and regulations in Tanzania. The approval was obtained from the ethics committee of the Muhimbili University of Health and Allied Sciences with ethical clearance no: MUHAS-REC-10-2019-062. Members of the study team held meetings with community, administrative, and religious leaders to explain the aims and activities of the study and sought community approval. Project CHWs then visited the households to explain the aim of the study, provided information sheets, and sought signed consent from parents or guardians of the children who were involved in the survey. The caregivers also provided the consent to participate in the socioeconomic survey.

### Statistical analysis

This was a baseline survey for seasonal malaria chemoprevention (SMC) study, thus sample size calculation was based on the estimations made for SMC, and is presented elsewhere [37].

Briefly, a samples size of 120 children in each of 20 wards selected was anticipated to provide a power of 80% and an alpha (type one error) of 0.05 with an attrition rate of 20% in explaining the variation of malaria incidence between the two districts. All the households with child/children were invited to participate in the socioeconomic status (SES) survey. Principal component analysis was used to estimate the scores for the socio-economic status of the households using household characteristics and assets. The SES scores of the first principal component were categorized into three groups of percentiles (low [0-39^th^], medium [40^th^-79^th^] and high SES [80^th^-100^th^]) for easy interpretation of results [36].

Data were collected electronically in open data kit (ODK) software using tablets computers. Spatial data (point coordinates for households and other features from the study areas) were collected during the household visit using Global Positioning System (GPS). Quality control and assurance of the data were maintained at all stages of data collection to archiving. Data were cleaned and analysed using STATA version 13 statistical packages. Categorical variables were presented in proportions and compared using *χ*2-tests while continuous variables were summarised as medians with associated range of inter-quartile range (IQR), means with standard deviations and 95% confidence intervals (95%CI). For continuous variables which were non-normal distributed, medians were compared using Wilcoxon rank-sum test while for normal distributed continuous variables, means were compared using *t*-test. A χ2-test was also used to assess the trend of continuous and categorical variables. Logistic regression analysis was used to assess the association between binary response variables (such as malnutrition indices) and risk factors in a univariate and multivariate models. Only variables that had a p-value <0.1 were included in the multivariable model. Variance inflation factor (VIF) was used to assess for multi-collinearity of independent variables, where a VIF<5 was considered a moderate correlation not requiring intervention. R-squared was used to assess how best the regression model fitted the data. A *p*-value < 0.05 was considered statistically significant.

## Results

### Baseline demographic and anthropometric characteristics

A total of 2242 children with median age of 27 months (range: 3.0–59.9) were involved in the study. 1539 (68.6%) of the study participants came from Masasi and 1169 (52.1%) of them were females. The baseline characteristics of the children are presented in Table 1 and supplementary data (S1). Compared to the WHO standard, children from the study area had higher BMI-for-age and weight-for-height but lower mean weight-for-age and height-for-age. Of the recruited children 48.9% (1064/2178) were undernourished. A significant proportion of children in Nanyumbu district (52.5%, 348/663) were undernourished compared to those in Masasi (47.3% (716/1515), (*x2* = 5.045, *p* = 0.025).

**Table 1:** Demographic and anthropometric characteristics of the study population by district.

Variable	Overall	Masasi	Nanyumbu	Test, *p-*value
Female sex, n (%)	1169 (52.1)	820 (53.3)	349 (49.6)	2.60^δ^, 0.110
Median age in years, (IQR)	2.32 (1.31–3.5)	2.25 (1.17–3.42)	2.25 (1.25–3.42)	-0.206^α^, 0.837
Mean weight, kg (SD)	11.42 (3.10)	11.47 (3.17)	11.31 (2.94)	1.1^β^, 0.267
Mean height, cm (SD)	81.97 (13.08)	82.20 (12.92)	81.46 (13.45)	1.25^β^, 0.212
MUAC (SD)	146.16 (12.52)	144.48 (12.89)	149.84 (10.81)	-9.592^β^, <0.001*
Mean BMI-for-age, (SD)	0.60 (1.60)	0.60 (1.55)	0.62 (1.69)	-0.243^β^, 0.808
Mean WAZ, (SD)	-0.60 (1.19)	-0.60 (1.23)	-0.66 (1.11)	1.56^β^, 0.119
Mean HAZ, (SD)	-1.56 (1.54)	-1.521 (1.54)	-1.64 (1.54)	1.83^β^, 0.067
Mean WHZ, (SD)	0.21 (1.38)	0.231 (1.37)	0.154 (1.40)	1.232^β^, 0.218
Underweight [WAZ< -2SD], n (%)	239 (11.1)	169 (11.30)	70 (10.6)	0.217^δ^, 0.641
Stunted [HAZ< -2SD], n (%)	900 (43.4)	608 (42.0)	292 (46.5)	3.624^δ^, 0.057
Wasted [WHZ< -2SD/ MUAC<115], n (%)	142 (7.4)	92 (6.8)	50 (8.9)	2.572^δ^, 0.109
Stunted and Wasted, n (%)	26 (1.2)	17 (1.1)	9 (1.3)	0.130^δ^, 0.719
Thin [BAZ< -2SD], n (%)	129 (6.1)	85 (5.8)	44 (6.9)	0.901^δ^, 0.343
Undernourished, n (%)	1064 (48.9)	716 (47.4)	348 (52.5)	5.045^δ^, 0.025*
Obese, n (%)	405 (19.3)	259 (17.7)	146 (22.8)	7.61^δ^, 0.006*

Δ: *X*2-test

α: Wilcoxon rank-sum-test

β: *t*-test

*significant at p<0.05.

### Relationship between anthropometric characteristics and gender

The relationship between anthropometric characteristics and the sex of the children is presented in Table 2. Overall, boys were significantly undernourished than girls. The prevalence of stunting and obesity was also significantly higher in boys than girls.

**Table 2:** Anthropometric characteristics in relation to the sex of study children.

Variable	Overall	Male	Female	Test, *p*-value
Mean weight, kg (SD)	11.42 (3.10)	11.57 (2.94)	11.28 (3.23)	2.19^β^, 0.028*
Mean height, cm (SD)	81.97 (13.08)	81.81 (12.62)	82.12 (13.50)	-0.56^β^, 0.576
Mean MUAC	146.16 (12.52)	146.74,(11.87)	145.62 (13.10)	2.11^β^, 0.035*
Mean BMI for age, (SD)	0.60 (1.60)	0.71 (1.62)	0.50 (1.58)	3.11^β^, 0.002*
Mean weight-for-age, (SD)	-0.60 (1.19)	-0.64 (1.20)	-0.57 (1.18)	-1.38^β^, 0.166
Mean height-for-age, (SD)	-1.56 (1.54)	-1.64 (1.56)	-1.48 (1.51)	-2.59^β^, 0.010*
Mean weight-for-height, (SD)	0.21 (1.38)	0.29 (1.43)	0.13 (1.34)	2.73^β^, 0.006*
Stunted, n (%)	900 (43.4)	468 (48.2)	432 (39.1)	17.44^δ^, <0.001*
Wasted, n (%)	142 (7.4)	65 (7.0)	77 (7.8)	0.426^δ^, 0.514
Underweight, n (%)	239 (11.1)	122 (11.9)	117 (10.4)	1.20^δ^, 0.273
Stunted and Wasted, n (%)	26 (1.2)	11 (1.0)	15 (1.3)	0.33^δ^, 0.569
Thin, n (%)	129 (6.1)	62 (6.2)	67 (6.1)	0.02^δ^, 0.887
Obese, n (%)	405 (19.3)	221 (22.1)	184 (16.7)	10.174^δ^, 0.001*
Undernourished, n (%)	1064 (48.9)	550 (53.0)	514 (45.0)	13.878^δ^, <0.001*

β: *t*-test

δ: *x*2-test

*significant at p<0.05.

Conversely, severe stunting, severe wasting and severe underweight occurred in 18.8% (391/2077), 2.6% (49/1917), 2.9% (63/2157) of the children. With the exception of severe stunting (*x2 = 21*.*6*, *<0*.*001*), the prevalence of severe wasting (*x2* = 3.01, *p* = 0.39) and severe underweight (*x2* = 2.52, 0.47*)* was not significantly different between boys and girls.

### Relationship between nutritional indices and age of children

**Fig 1:**
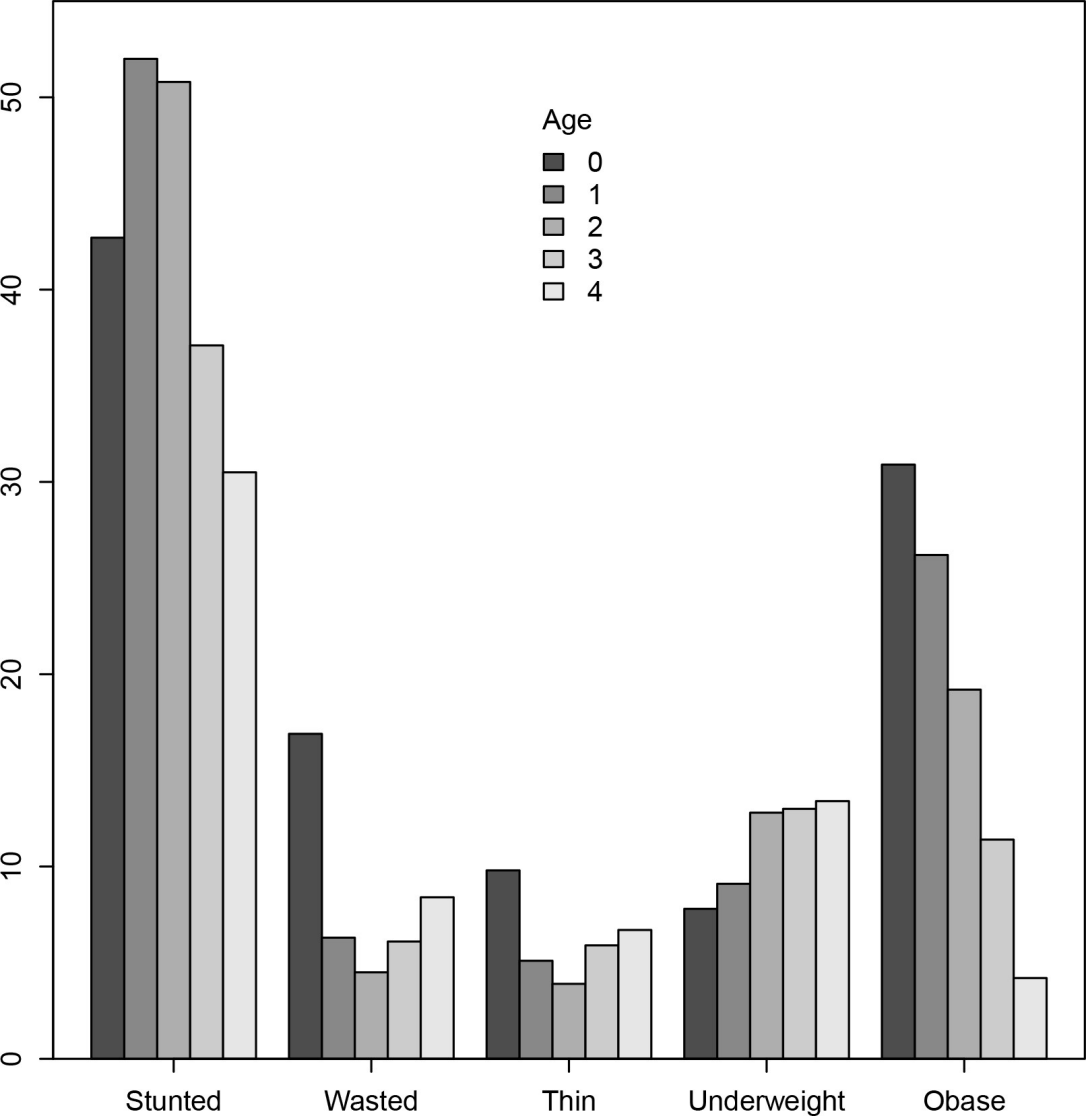
Nutritional characteristics of the study population in relation to age groups.

Table 3 presents the relationship between nutritional indices and age groups of the children. The prevalence of stunting was highest among children aged 1 year, whereas that of wasting, thinness and obesity was highest in those aged less than 1 year, while that of underweight was highest in those aged 4 years. The trend of prevalence of stunting (trend *x*2 = 53.86, *p*<0.001), wasting (trend *x*2 = 36.53, *p*<0.001), thinness (trend *x*2 = 14.42, *p* = 0.006) and obesity (trend *x*2 = 69.11, *p*<0.001) was decreasing significantly with an increasing age, whereas that of underweight (trend *x*2 = 11.43, *p* = 0.022) was increasing with increasing age. It was interesting to note that underweight and obesity had linear relationship with age; the increase in age was associated with the increase in the risk of underweight and vice versa for the obesity, Fig 1. Contrarily, stunted, wasted and thin showed quadratic relation with age, whereby while the risk of stunted was elevated at the age of 1–2 years, the risk of being wasted and thin was lowest at the age of two years, Fig 1.

**Table 3:** Nutritional characteristics of the study population in relation to age groups.

	Nutritional characteristic				
Age group	Stunted, n(%)	Wasted, n (%)	Underweight, n (%)	Thin, n (%)	Obese, n (%)
0	172 (42.7)	37 (16.9)	33 (7.8)	39 (9.8)	130(30.9)
1	245 (52.0)	30 (6.3)	45 (9.1)	25 (5.1)	127 (26.2)
2	229 (50.8)	21 (4.5)	60 (12.8)	18 (3.9)	87 (19.2)
3	150 (37.1)	25 (6.1)	54 (13.0)	24 (5.9)	47 (11.4)
4	104 (30.5)	29 (8.4)	47 (13.4)	23 (6.7)	14 (4.2)
*x*^2^_(4d.f)_, *p*-value	53.86, <0.001	36.53, <0.001	11.43, 0.022	14.42, 0.006	116.39, <0.001

### Relationship between nutritional status and malaria infection

Fourteen percent (312/2179) of the children had malaria infection. Only 15.8% (168/1064) of the undernourished children had malaria infection. The relationship between nutritional status and malaria infection is presented in Table 4. None of the nutritional indices was significantly associated with malaria infection in the study population (p>0.05), although children who were wasted (10.1% vs. 6.9%, *p* = 0.06) and thin (8.5 vs. 5.7, *p =* 0.06) had slightly higher risk of malaria infection.

**Table 4:** Nutritional status in relation to malaria infection.

Nutritional status	Malaria infection status		*x*2-test, *p*-value
	mRDT positive, n (%)	mRDT negative, n (%)	
Stunted	135 (45.8)	767 (42.9)	0.81, 0.370
Wasted	29 (10.1)	113 (6.9)	3.58, 0.058
Stunted and wasted	7 (2.1)	23 (1.4)	0.78, 0.378
Underweight	40 (12.9)	199 (10.8)	1.22, 0.269
Thin	26 (8.5)	103 (5.7)	3.54, 0.06
Obese	58 (14.3)	247 (14.5)	0.01, 0.908
Undernourished, n (%)	168 (53.8)	896 (48.0)	3.63, 0.057

### Relationship between socio-demographic factors and undernourishment

In a univariate analysis district, sex of the children, age group anaemia and malaria were the factors found to be associated with undernourishment. The multivariate analysis on the other hand indicated sex, age group and anaemia were significantly associated with undernourishment (p<0.05), while district and malaria infection were marginally (p≤0.06) associated with undernourishment. The relationship between socio-demographic factors and undernourishment is presented in Table 5.

**Table 5:** Relationship between socioeconomic factors and undernutrition using logistic regression models.

Variable	Univariate	Multivariate*
	OR (95%CI), *p*-value	aOR (95%CI), *p*-value
District—Masasi	1	1
• Nanyumbu	1.25 (1.05–1.5), 0.014	1.20 (0.99–1.44), 0.062
Sex of child–Male	1	1
• Female	0.72 (0.61–0.85), <0.001	0.75 (0.63–0.89), 0.001
Number of people: 1–5	1	
• More than 5	1.10 (0.86–1.4), 0.451	
Number of <5 children: One	1	
• More than one	1.08 (0.83–1.41), 0.56	
Marital status of household head		
• Single	1	
• Couple	0.84 (0.67–1.05), 0.125	
• Others	0.97 (0.60–1.5), 0.902	
Education of head: Secondary	1	
• None	1.09 (0.68–1.74), 0.727	
• Primary	1.20 (0.85–1.70), 0.307	
Occupation of head: Farmers	1	
• Petty trader	0.79 (0.52–1.19), 0.257	
Household SES of score category		
Upper [80^th^ -100^th^]	1	
Low [0-39^th^]	1.03 (0.78–1.36), 0.84	
Middle [40^th^-79^th^]	0.96 (0.73–1.27), 0.764	
Age group of children (years)		
• 4	1	
• 0	1.72 (1.30–2.29), <0.001	1.63 (1.20–2.21), 0.002
• 1	2.20 (1.66–2.90), <0.001	2.17 (1.61–2.92), <0.001
• 2	1.91 (1.44–2.53), <0.001	1.87 (1.39–2.50), <0.001
• 3	1.31 (0.98–1.75), 0.069	1.32 (0.98–1.79), 0.066
Anaemia in children: Normal	1	1
• Anemic	1.45 (1.22–1.73), <0.001	1.22 (1.01–1.47), 0.039
mRDT negative	1	1
• mRDT positive	1.26 (1.01–1.58), 0.044	1.26 (0.99–1.59), 0.058

OR: Odds ratio

aOR: Adjusted odds ratio

CI: Confidence Interval

*Mean VIF = 1.63, range (1.25–2.45).

## Discussion

Malnutrition and malaria are among the major public health challenges in Tanzania. This study assessed the situation of malnutrition among underfive children in Masasi and Nanyumbu districts, its interaction with malaria infection and how it is influenced by socioeconomic factors. Nearly half of the recruited children were undernourished. Similar high prevalence of undernutrition has been reported in Cameroon [15,38]. The prevalence of undernutrition was significantly higher in Nanyumbu than Masasi district. Of the undernutrition indices stunting was the most prevalent, and it was significantly more prevalent in Nanyumbu than Masasi district. Higher prevalence of stunting has also been reported in other parts of Tanzania [38,39], and in other countries [3,15,25,34]. The prevalence of stunting in this study population was however much higher than the national average of 31.8% reported in 2018 [30]. Other indices namely wasting and thinness were more prevalent in Nanyumbu district, however underweight was more prevalent in Masasi district. On the other hand boys were more undernourished than girls, a finding similar to that reported in Nigeria [3]. In addition, stunting was significantly more prevalent in boys than girls. Studies in other parts of Tanzania [39,40], and in Cameroon [1], have also indicated higher prevalence of stunting in males than in females. It is not clear why in this study more males were undernourished and stunted than females. However, other studies have linked it with more boys given complementary food than girls [38,41], early weaning for boys [38,42], and children behavior where girls might stay closer to home than boys and have more access to food being cooked [43]. Severe stunting occurred in about 19% of the children, whereas severe wasting and severe underweight occurred only in 2.6% and 2.9% of the children, respectively. The prevalence of severe stunting in this study population was much less than that reported in other parts of Tanzania [38], but was nearly four times higher than that reported in Nigeria [1] and Ghana [12]. Presence of severe stunting in a population is a cause of concern since it indicates chronic malnutrition, and results from poor nutrition in early childhood that leads to failure to grow both physically and mentally [12,14]. Proper nutrition is crucial for enhancing brain function and improving learning [44]. Childhood with early-onset persistent stunting can have long-term effects on mental development, academic performance, economic productivity in adulthood and maternal reproductive outcomes [43,45,46]. On the other hand, other undernutrition indices including wasting, underweight and thinness were not significantly different between genders. Contrary to the findings in this study, a study in Rwanda has found underweight to be more prevalent in males [25].

Stunting was highest in 1 year old age group followed by 2 years old group, whereas wasting, thinness and obesity were highest among children aged less than 1 year group, and underweight was highest among children in 4 years age group. Similar findings have been reported in other countries [3,24] However, whereas stunting, wasting, thinness and obesity were decreasing with increasing age, underweight was increasing with increasing age. The variation in prevalence of undernutrition indices with age groups may be explained by the availability of nutrients at these different stages. As infants grow especially around 1 year old the nutrients obtained from breast milk becomes inadequate, whereas during weaning the infants fail to eat adequately, and also probably the alternative foods may not have adequate nutrients thus predisposing them to stunting. Inadequate nutrients may also lead to underweight, and if continues may lead to the increased prevalence of underweight and decreasing prevalence of obesity with increasing age. On the other hand, as the child grow and start to adopt to weaning foods they get enough nutrients to support their growth, and this may explain the decreasing prevalence of stunting, wasting and thinness with increasing age.

Only about 16% of the undernourished children had malaria infection. Furthermore, all the malnutrition indices including stunting, wasting, underweight and obesity had no association with malaria infection. Similar to the findings in this study, other studies have also found stunting [14,16,22], underweight [16,17,22,23], and wasting [16,17,23] to have no association with malaria infection. Nevertheless, some studies have found a significant association between stunting [1,3,17,18] and underweight [1,12] on one hand with malaria infection on the other hand. However, intriguingly although not statistically significantly different to the none-undernourished children, more than half of the malaria infected children were also undernourished. Furthermore, whereas in the univariate analysis malaria infection was not associated with undernutrition, in the multivariate analysis the infection was statistically significantly associated with undernutrition. A strong association between undernutrition and malaria infection has also been reported in other studies [5,15,24], with some studies indicating undernutrition to increase susceptibility to malaria infection [14,15], whereas others found malaria to increases the risk of malnutrition [6]. However, other studies have indicated lack of association between malaria infection and undernutrition [17,25,47]. Undernutrition suppresses the body immunity [10–12], therefore, increasing the risk of infection including malaria [14,15]. Likewise, malaria infection leads to individual energy loss with reduced productivity, and in a long run leads to a spiral of malnutrition, infections and poverty [10,11].

In the study districts especially Nanyumbu, male gender, age group of children below 3 years and anaemia were the only other factors associated with malnutrition. Other studies have also found male gender [1,3,15,26,27] location, age group and anaemia to be associated with malnutrition [1]. However, in this study other factors including household income level, caregiver education level, caregiver marital status, number of household occupants and household number of underfive children were not associated with undernutrition. Similar findings have been reported in another study [24]. Contrary to the findings in this study, other studies have found low household level of income [3,5,15,25–28], lack of formal education of caregivers [3,15,28], source of water for domestic use and its distance [27,29], extended larger families [26], and occupation of the mother [5,15,26,28] to influence malnutrition in underfive children. This clearly shows that the level of influence the socioeconomic factors impose on malnutrition varies from one setting to another, probably also indicating that other factors than socioeconomic are influencing the occurrence of malnutrition.

## Conclusion

Undernutrition was highly prevalent in the study population and was influenced by sex, age, anaemia and malaria infection. More emphasis is needed to address the malnutrition problem especially stunting in Masasi and Nanyumbu districts.

## Supporting information

S1 File Anthropometric measurements by age and sex. (XLS)

## Abbreviations

ACT: Artemisinin based combination therapy
CHWs: Community Health Workers
CI: Confidence Interval
GPS: Global Positioning System
Hb: Haemoglobin
IQR: Inter-quartile range
ITN: Insecticide Treated Nets
mRDT: Malaria Rapid Diagnostic Test
NIMR: National Institute for Medical Research
OKD: Open Data Kit
SD: Standard deviation
SMC: Seasonal Malaria Chemoprevention
SSA: Sub-Saharan Africa
WBC: White Blood Cells
